# Case Report: Cholestatic liver injury during atezolizumab therapy in EGFR-mutant combined small cell lung cancer with possible underlying primary biliary cholangitis

**DOI:** 10.3389/fonc.2026.1979503

**Published:** 2026-10-27

**Authors:** Yingqiong Zhu, Jin Xiong, Chuan Tan, Benxu Tan, Yusheng Huang, Ganchao Zhao

**Affiliations:** 1 Chongqing Three Gorges Medical College Affiliated Fengjie Hospital, Chongqing, China; 2 Department of Radiation Medicine, School of Basic Medical Sciences, Chongqing Medical University, Chongqing, China; 3 The First Affiliated Hospital of Army Medical University (Southwest Hospital), Chongqing, China; 4 Department of Cancer Center, The Second Affiliated Hospital of Chongqing Medical University, Chongqing, China

**Keywords:** atezolizumab, cholestatic liver injury, combined small cell lung cancer, EGFR mutation, immune checkpoint inhibitor-associated cholangiopathy, immune-related adverse events, primary biliary cholangitis

## Abstract

**Background:**

Combined small cell lung cancer (C-SCLC) may coexist with EGFR driver mutations. For extensive-stage disease, PD-L1 inhibitor plus platinum–etoposide is first-line. Immune checkpoint inhibitor (ICI)-associated cholangiopathy is a rare immune-related adverse event hard to distinguish from pre-existing autoimmune biliary disease such as primary biliary cholangitis (PBC) when baseline serology and histology are unavailable.

**Case presentation:**

A 68-year-old male former smoker presented with an incidentally detected right upper lobe mass. CT-guided percutaneous biopsy showed C-SCLC with a small adenocarcinoma component, neuroendocrine marker positivity, and loss of Rb, and next-generation sequencing revealed an EGFR exon 21 L858R mutation (mutant allele frequency 60.78%). The disease was classified as extensive-stage C-SCLC. First-line etoposide, carboplatin, and atezolizumab (four cycles) achieved partial response; alkaline phosphatase rose from mildly elevated baseline (156 U/L) to 532 U/L and gamma-glutamyl transferase from 87 to 317 U/L, with mild transaminase elevation and normal total bilirubin, a cholestatic pattern (CTCAE v5.0 grade 2). AMA-M2 was positive, raising the possibility of a previously unrecognized PBC background. Imaging showed no biliary dilatation, steatosis, or advanced fibrosis, but magnetic resonance cholangiopancreatography, liver biopsy, and pre-treatment autoantibody testing were not performed. Atezolizumab was stopped and ursodeoxycholic acid continued without high-dose corticosteroids. The cholestatic enzymes fell thereafter, but neither an underlying PBC nor a contribution from superimposed ICI-related biliary injury could be established. Consolidative thoracic radiotherapy and concurrent maintenance with furmonertinib and oral etoposide followed. At the last follow-up on July 28, 2026, partial response was maintained.

**Conclusion:**

This patient with EGFR-mutant C-SCLC was managed with SCLC-directed chemo-immunotherapy then maintenance with EGFR tyrosine kinase inhibitor after cholestatic liver injury. AMA-M2 positivity with pre-existing cholestatic abnormalities raises the possibility of an underlying PBC, but without pre-treatment serology, magnetic resonance cholangiopancreatography, or liver biopsy, neither a definite PBC background nor a definite ICI-related cholangiopathy could be established. Cholestatic liver injury during immunotherapy warrants evaluation for autoimmune biliary disease and drug-related biliary injury.

## Introduction

Combined small cell lung cancer (C-SCLC), comprising small cell and non-small cell components, accounts for 5%–28% of small cell lung cancers and is molecularly heterogeneous (1, 2). EGFR mutations, more typical of lung adenocarcinoma, can coexist with small cell morphology (3, 4). For extensive-stage disease, first-line treatment combines platinum–etoposide with a PD-L1 inhibitor, supported by the phase III IMpower133 trial (5) and meta-analyses (6). When an EGFR driver is present, the optimal sequencing of chemotherapy, immunotherapy, and EGFR tyrosine kinase inhibitors (TKIs) remains debated, because evidence for EGFR-TKI efficacy in C-SCLC remains limited and is largely case-based (7, 8).

Immune checkpoint inhibitors (ICIs) cause a spectrum of immune-related adverse events, and ICI-related hepatotoxicity includes hepatocellular and cholestatic patterns. Immune-mediated hepatitis predominantly affects hepatocytes, whereas ICI-associated cholangiopathy is a distinct cholestatic phenotype with alkaline phosphatase and gamma-glutamyl transferase predominance, delayed onset, and limited glucocorticoid responsiveness (9–12). This creates diagnostic difficulty when cholestatic injury occurs with susceptibility to autoimmune biliary disease. Primary biliary cholangitis (PBC) can be asymptomatic or present with normal or mildly elevated alkaline phosphatase (13–15); in adults with otherwise unexplained cholestasis, the diagnosis rests on an elevated alkaline phosphatase together with antimitochondrial antibody positivity, and liver biopsy is not required unless PBC-specific antibodies are absent or coexistent disease is suspected (16). Rare ICI-era reports describe PBC-like or granulomatous liver injury, raising the question of whether checkpoint blockade unmasks a pre-existing autoimmune background or generates a *de novo* PBC-like phenotype (17–19).

A 68-year-old man with EGFR-mutant C-SCLC developed cholestatic liver injury during first-line atezolizumab plus etoposide–platinum. AMA-M2 positivity in a patient with pre-treatment cholestatic enzyme abnormalities raised the possibility of an underlying PBC, yet absent pre-treatment autoantibody testing, magnetic resonance cholangiopancreatography, and liver biopsy left both the presence of PBC and its relationship to ICI exposure uncertain. We report this case to examine the diagnostic distinction between cholestatic liver injury occurring during atezolizumab therapy in a patient with possible underlying PBC and *de novo* ICI-related PBC-like cholangiopathy, and to describe the clinical course after atezolizumab discontinuation.

## Case presentation

A 68-year-old male former smoker (20 pack-years) was admitted on May 6, 2025. He had been asymptomatic, and the right upper lobe lung mass had been detected during a routine health examination; his Eastern Cooperative Oncology Group performance status was 0. Admission carcinoembryonic antigen was elevated (16.29 ng/mL; reference 0.00–4.50), as were cytokeratin 19 fragment (3.74 ng/mL), tissue polypeptide antigen (187.20 U/L), and pro-gastrin-releasing peptide (83.91 pg/mL); neuron-specific enolase and squamous cell carcinoma antigen were within reference range and ferritin was below it (8.20 ng/mL). Baseline thyroid-stimulating hormone was mildly elevated (7.79 µIU/mL) with normal free and total thyroid hormones. Physical examination revealed no remarkable positive findings. Relevant medical history comprised hypertension of 20 years’ duration and subtotal gastrectomy 30 years earlier for peptic ulcer disease; the patient had no known autoimmune disease, viral hepatitis, or biliary disease, and family history was non-contributory. Baseline liver function tests showed a cholestatic-predominant pattern with alkaline phosphatase 156 U/L and gamma-glutamyl transferase 87 U/L (both mildly elevated; institutional upper limits of normal 125 and 60 U/L, respectively), normal alanine and aspartate aminotransferase, and normal total bilirubin (11.2 µmol/L). Baseline contrast-enhanced chest computed tomography showed a 56 × 35 mm primary soft-tissue mass in the right upper lobe anterior segment with adjacent limited atelectatic change, together with bilateral hilar and mediastinal nodal disease. Contralateral left hilar nodal involvement (station 10L) supported N3 disease. A separate approximately 28 × 22 mm lesion in the left upper lobe anterior segment near the hilum, with an ill-defined margin, mild enhancement and associated partial bronchial occlusion, was clinically considered a solitary contralateral pulmonary metastasis, establishing M1a disease. The clinical stage was therefore cT3N3M1a (AJCC 8th edition; stage IVA), corresponding to extensive-stage small cell lung cancer with a clinically described low metastatic burden. Abdominal computed tomography, brain magnetic resonance imaging and bone scintigraphy showed no extrathoracic metastases. CT-guided percutaneous lung biopsy demonstrated combined small cell lung cancer with a focal adenocarcinoma component (**Figure 1**). Immunohistochemistry showed TTF-1, synaptophysin, CD56, chromogranin A, and INSM1 positivity with loss of Rb, and the Ki-67 index was approximately 45%. Next-generation sequencing of the composite tumor specimen identified an EGFR exon 21 L858R mutation (c.2573T>G; mutant allele frequency 60.78%) with microsatellite stability and no concomitant ALK/ROS1/RET rearrangements or KRAS/BRAF/NRAS/PIK3CA/ERBB2/MET mutations. Component-level sequencing was not performed, so the cellular origin of the mutation cannot be assigned to either histologic component.

**Figure 1:**
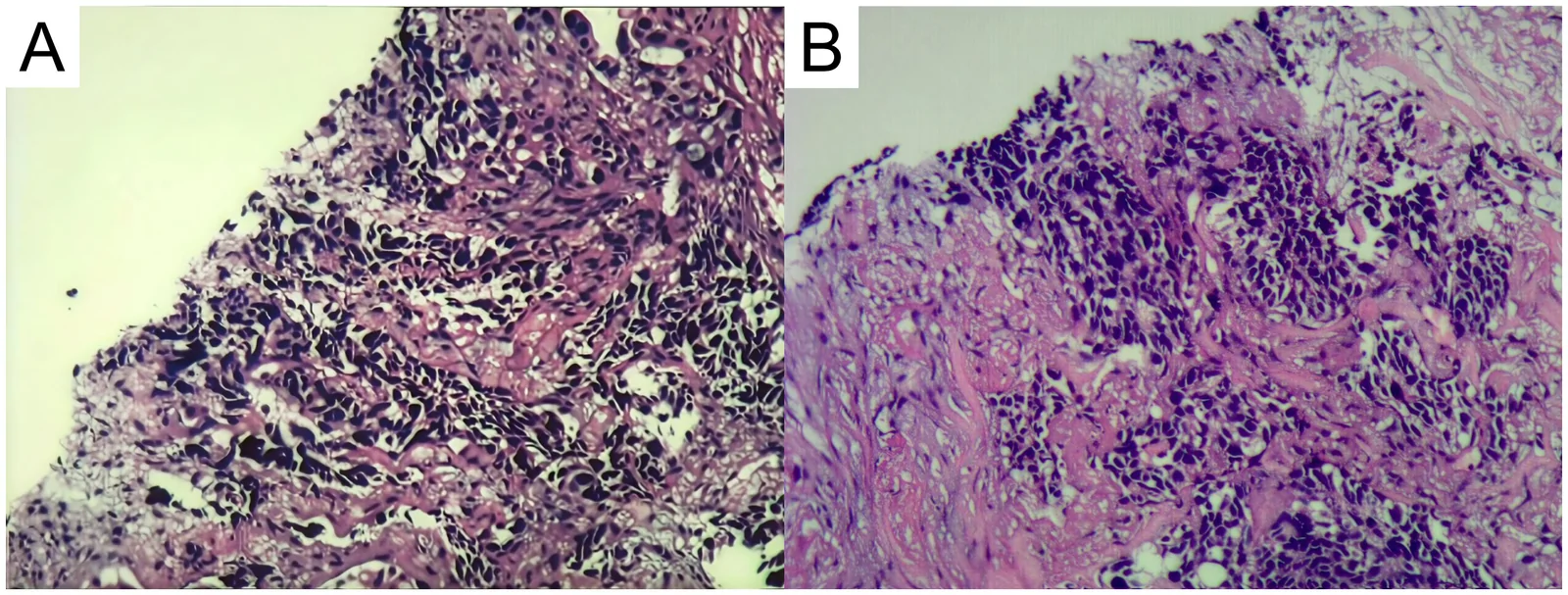
Histopathological findings of combined small cell lung cancer. **(A)** Representative hematoxylin and eosin (H&E)-stained section showing predominantly small-cell carcinoma morphology, characterized by densely packed small hyperchromatic cells with scant cytoplasm and a high nuclear-to-cytoplasmic ratio. **(B)** Representative H&E-stained section containing a focal adenocarcinoma component within the combined tumor, with associated desmoplastic stromal reaction. Original magnification, ×200. The immunohistochemical findings (TTF-1, synaptophysin, CD56, chromogranin A, INSM1 positivity, and loss of Rb) are reported in the Case presentation; no immunohistochemical image is shown in this figure. No scale bar is shown because the available source images are not calibrated.

First-line therapy with etoposide, carboplatin, and atezolizumab (four cycles) achieved partial response (**Figure 2**); systemic disease control was sustained and no new distant metastatic lesions appeared during first-line treatment. After four cycles, alkaline phosphatase rose from 156 to 532 U/L and gamma-glutamyl transferase from 87 to 317 U/L, with only mild transaminase elevation and normal total bilirubin, a cholestatic injury pattern corresponding to CTCAE version 5.0 grade 2 (**Figure 3**). AMA-M2 was positive (62.6 RU/mL) when first tested after four cycles of therapy; pre-treatment AMA or AMA-M2 results were not available. Liver ultrasound showed hepatic cysts, the largest 46 × 38 mm, with no intrahepatic or extrahepatic biliary dilatation, and transient elastography on September 15, 2025, showed a controlled attenuation parameter of 160 dB/m and liver stiffness of 7.0 kPa, consistent with neither significant steatosis nor advanced fibrosis. Magnetic resonance cholangiopancreatography, endoscopic retrograde cholangiopancreatography, and liver biopsy were not performed, so the injury could not be confirmed histologically and its biliary subtype could not be defined.

**Figure 2:**
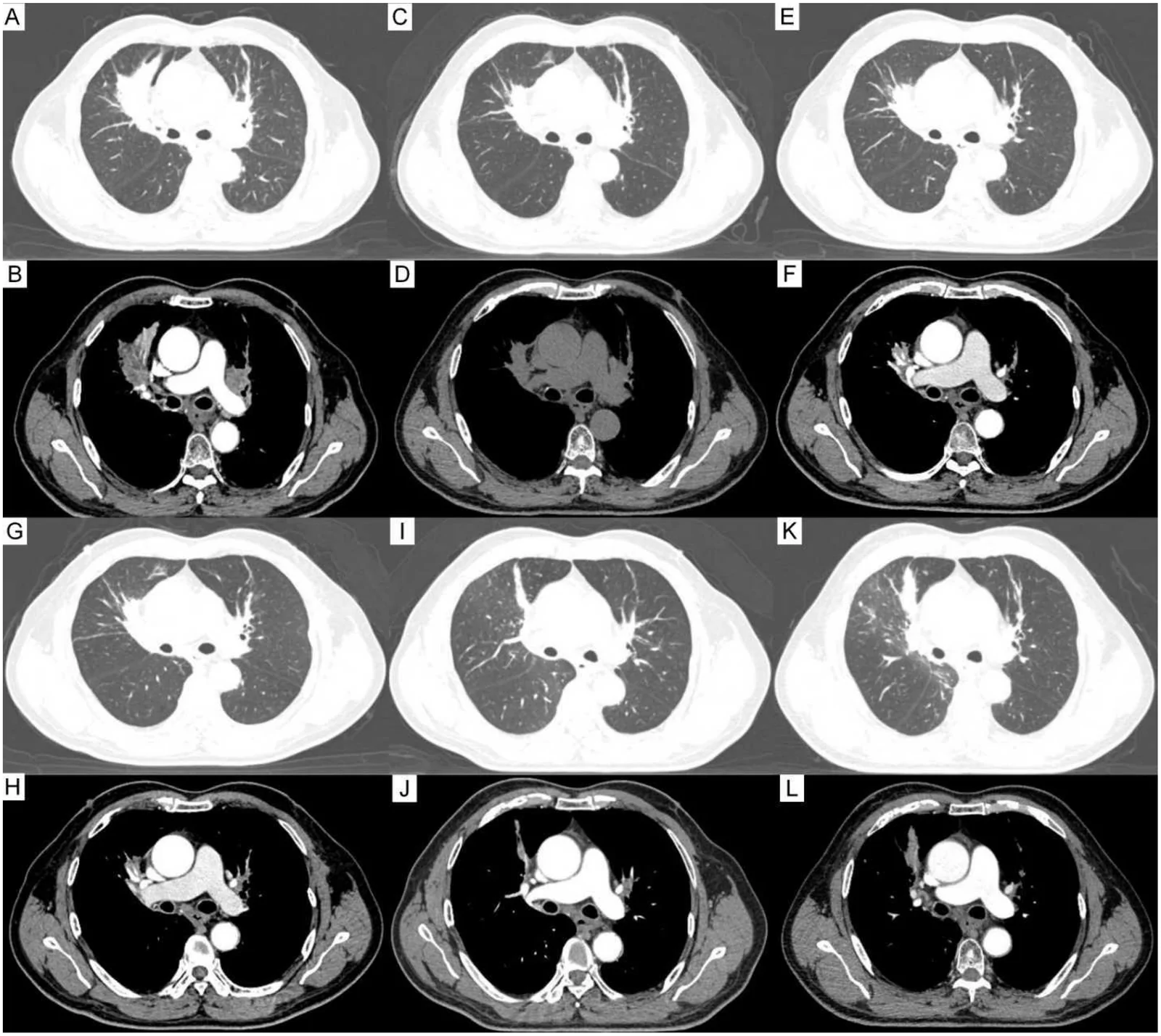
Radiological response assessment during treatment. Twelve computed-tomography images **(A–L)** are arranged in three columns and four rows; rows 1 and 3 show lung-window images, whereas rows 2 and 4 show the corresponding mediastinal-window images. For each pair, the first panel shows the lung window and the second shows the mediastinal window. A/B = baseline (6 May 2025); **(C, D)** = after two cycles of etoposide–carboplatin–atezolizumab (30 June 2025); **(E, F)** after four cycles (20 August 2025); **(G, H)** follow-up/restaging (7 November 2025); **(I, J)** post-consolidative thoracic radiotherapy follow-up (26 March 2026); **(K, L)** later follow-up/pre-prophylactic-cranial-irradiation assessment (25 May 2026). The right upper lobe lesion and nodal involvement regressed during treatment.

**Figure 3:**
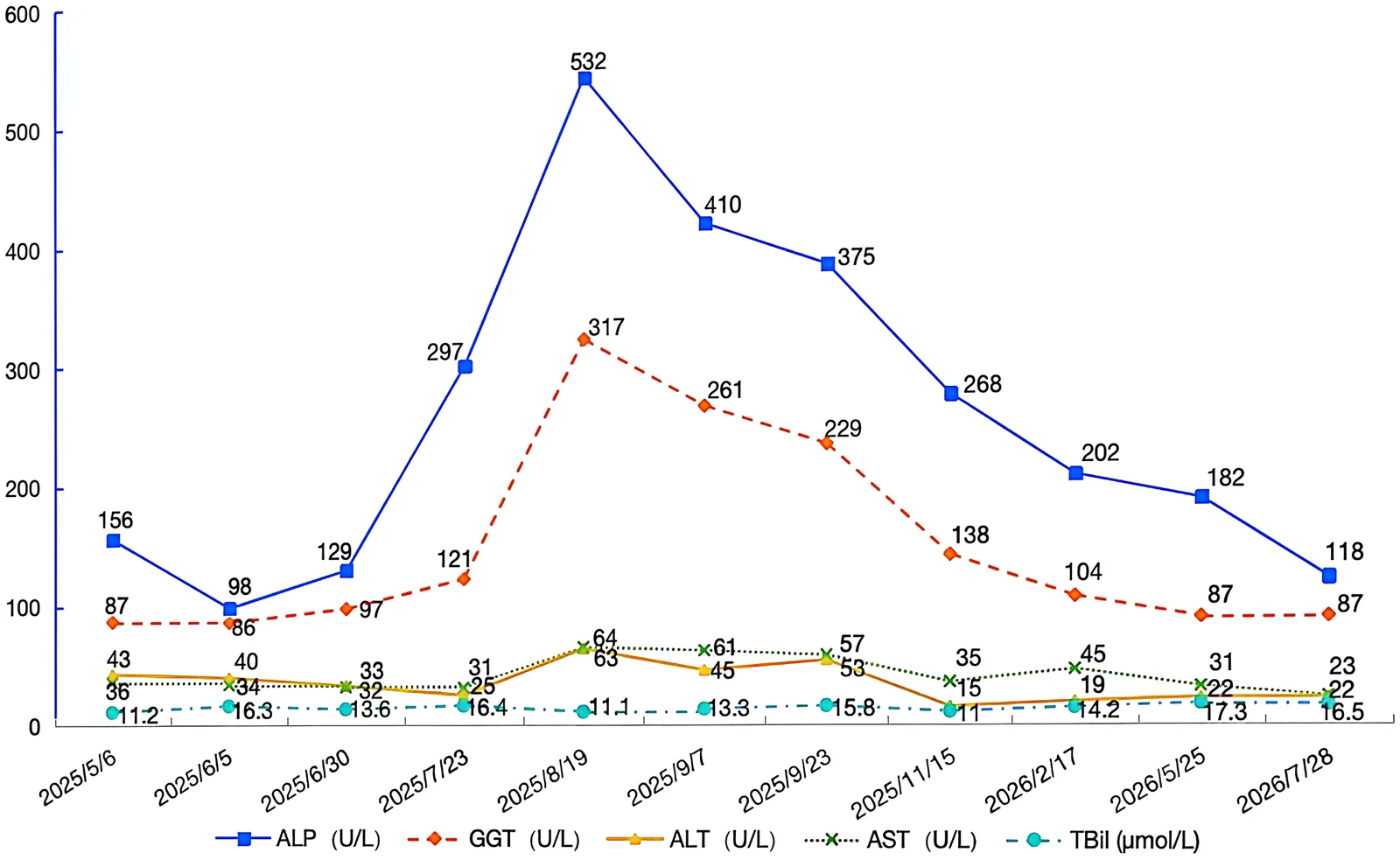
Dynamic changes in liver biochemistry during treatment, shown as a single shared-axis line chart. ALP, alkaline phosphatase; GGT, gamma-glutamyl transferase; ALT, alanine aminotransferase; AST, aspartate aminotransferase; TBil, total bilirubin. All five analytes are displayed on a common numerical axis for visualization; enzyme activities are expressed in U/L and total bilirubin in µmol/L. Institutional upper limits of normal: ALP 125 U/L; GGT 60 U/L; ALT 50 U/L; AST 40 U/L; TBil 28.0 µmol/L. Eleven serial laboratory measurements are shown at their labelled examination dates from 6 May 2025 to 28 July 2026. The numerical value of each measurement is printed beside its own point, to one decimal place for total bilirubin and as an integer for the enzyme activities. No treatment events, no treatment bands and no clinical timeline are shown in this figure; they are presented in **Figure 4**.

Hypothyroidism developed with markedly elevated thyroid autoantibodies (thyroglobulin antibody >4000 IU/mL, thyroid peroxidase >600 IU/mL) and required levothyroxine; thyroid function evolved from a mildly elevated baseline TSH (7.79 µIU/mL) to thyrotoxicosis and then overt hypothyroidism (TSH 90.1 µIU/mL), a course consistent with ICI-associated thyroiditis. Available viral hepatitis testing was negative. Serum ceruloplasmin was elevated (0.394 g/L). Immunoglobulin G and A measured after liver injury were elevated (IgG 17.5 g/L, IgA 5.64 g/L) and IgM was within reference range, findings consistent with broader immune activation but detected only after the liver injury. No clinical or imaging evidence supported biliary obstruction or tumor-related hepatic involvement.

Atezolizumab was discontinued and ursodeoxycholic acid (UDCA) was continued. High-dose corticosteroids were withheld following multidisciplinary assessment because the liver injury was cholestatic-predominant with normal bilirubin and PBC-related serologic findings were present. Maintenance therapy with furmonertinib 80 mg once daily, directed at the EGFR L858R alteration, and oral etoposide 50 mg once daily for 21 days of each 4-week cycle (capsule strength 25 mg) was initiated on 19 August 2025, 26 days after the last atezolizumab dose (24 July 2025). Dose-intensified consolidative thoracic radiotherapy (60 Gy in 30 fractions of 2 Gy, intensity-modulated radiotherapy, to the right lung lesion and mediastinal regions) was delivered from 12 January to 24 February 2026 concurrently with this maintenance therapy and was completed as planned without clinically significant interruption. Approximately 3 months after completion of thoracic radiotherapy, follow-up chest computed tomography on 25 May 2026 showed sustained disease control. After contrast-enhanced brain magnetic resonance imaging on 26 May 2026 confirmed the absence of brain metastases, prophylactic cranial irradiation (25 Gy in 10 fractions) was delivered from 28 May to 11 June 2026 following an individualized benefit-risk assessment and was completed as planned. Both radiotherapy courses were completed without treatment interruption; mild radiation-related esophagitis, pneumonitis and fatigue were documented, and no formal CTCAE grading of these radiotherapy-related toxicities was available. Oral etoposide was still being taken at the last follow-up on 28 July 2026, at which time partial response was maintained. Adherence to furmonertinib and oral etoposide was good, and no clear treatment-related adverse events, including grade ≥ 3 events, were observed during the maintenance period. No relevant psychosocial history was reported. The longitudinal liver-biochemistry trajectory is shown in **Figure 3**, and the integrated clinical timeline is presented in **Figure 4**.

**Figure 4:**
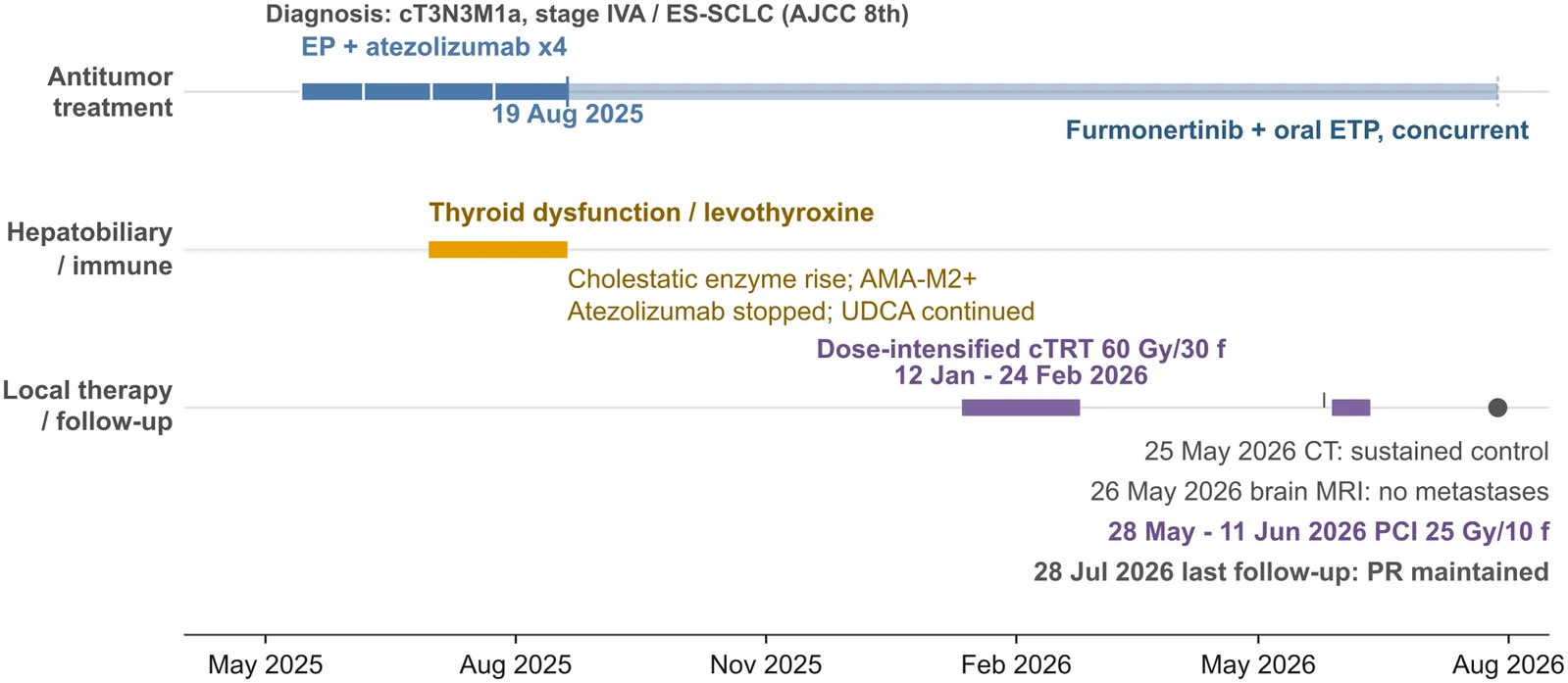
Integrated clinical timeline for the same patient, combining the antitumor treatment course, the hepatobiliary and immune events, and the local therapy and follow-up on a single continuous time axis. The clinical stage at diagnosis was cT3N3M1a (AJCC 8th edition; stage IVA), corresponding to extensive-stage small cell lung cancer. Solid horizontal bands denote treatment periods documented at both ends; a dashed band edge indicates that the corresponding boundary is not documented in the available record. The concurrent maintenance regimen of furmonertinib and oral etoposide began on 19 August 2025; because this treatment was still ongoing at the last documented follow-up on 28 July 2026 and the subsequent termination date is unknown, the right end of the maintenance band remains open, so that no treatment is implied beyond the available record. The short vertical breaks within the first band mark the individual cycles of etoposide, carboplatin and atezolizumab. Dose-intensified consolidative thoracic radiotherapy (60 Gy in 30 fractions) was delivered from 12 January to 24 February 2026, and prophylactic cranial irradiation (25 Gy in 10 fractions) from 28 May to 11 June 2026; both courses were completed as planned. Follow-up chest computed tomography on 25 May 2026 showed sustained disease control approximately 3 months after completion of thoracic radiotherapy, and contrast-enhanced brain magnetic resonance imaging on 26 May 2026 showed no brain metastases. At the last follow-up on 28 July 2026, partial response was maintained. EP, etoposide plus platinum; ETP, etoposide; cTRT, consolidative thoracic radiotherapy; PCI, prophylactic cranial irradiation; UDCA, ursodeoxycholic acid; AMA-M2, anti-mitochondrial antibody M2; PR, partial response. Furmonertinib and oral etoposide were administered concurrently and are shown as a single band. Events are placed at their documented dates. Liver-enzyme values are not shown here; the biochemical trajectory is presented in **Figure 3**.

## Discussion

### Treatment rationale: why EP plus atezolizumab was selected as first-line therapy for EGFR-mutant C-SCLC

Although molecularly distinct from pure small cell lung cancer, C-SCLC is generally managed with small cell lung cancer protocols (1, 2). EGFR mutations are rare in C-SCLC but reported, including the same L858R mutation with small cell morphology as in this patient (3, 4). In extensive-stage disease, first-line PD-L1 inhibitor plus platinum–etoposide improved overall survival (median 12.3 vs. 10.3 months; hazard ratio 0.70, 95% confidence interval 0.54–0.91) and progression-free survival (median 5.2 vs. 4.3 months) in IMpower133 (5), corroborated by meta-analyses (6).

Whether EGFR-targeted therapy should be preferred when a driver is present remains unclear because evidence is limited (7, 8). Identical EGFR mutations across components of some C-SCLC tumors (3, 4) caution against assigning the alteration solely to adenocarcinoma. Thus, SCLC-directed chemo-immunotherapy followed by an EGFR TKI reflected the absence of prospective sequencing evidence, not proven superiority.

Composite-tumor sequencing identified EGFR L858R at 60.78% variant allele frequency, but component-level origin remains unknown. The patient therefore received the established extensive-stage etoposide-platinum-atezolizumab regimen, followed by EGFR-directed therapy for the actionable alteration.

### ICI-associated cholangiopathy: a cholestatic immune-related adverse event distinct from hepatitis

Systematic reviews of immune-related cholangitis describe a median alkaline phosphatase of 1328 U/L with only mild transaminase elevation, together with small-duct, large-duct, and mixed subtypes and CD8+ T-cell infiltration on histology (9, 10). Median onset in French pharmacovigilance data was 5.7 months (11). In a 530-patient cohort, imaging-defined sclerosing cholangitis occurred in 0.8% versus 1.5% with hepatitis, and the two groups differed in glucocorticoid use and recovery (12).

Complete glucocorticoid response was only 11.5% (3 of 26) (10), and median recovery to grade 1 was 102.5 versus 36 days (12). Histologically, ICI-related liver injury can present as hepatitis, cholangitis, or mixed patterns distinguishable from classic autoimmune liver disease (20).

In this patient, alkaline phosphatase and gamma-glutamyl transferase were already mildly abnormal before atezolizumab and rose further during treatment, with mild transaminase elevation and normal bilirubin. This cholestatic pattern is compatible with, but not diagnostic of, ICI-related biliary injury.

### Differential diagnosis and attribution of cholestatic liver injury: comparing competing explanations

Comparative studies distinguish ICI-related liver injury histologically from autoimmune hepatitis and drug-induced liver injury by granuloma formation and altered CD4/CD8 ratios (21), and drug-induced bile-duct injury is divided into small-duct and large-duct types (22). Malignant biliary infiltration can fully mimic cholangiopathy (23).

Biochemistry alone cannot distinguish chemotherapy-related injury, malignant infiltration, PBC progression or autoimmune hepatitis-PBC overlap, IgG4-related cholangitis, and obstruction, which require different management.

Sustained partial response made progressive biliary infiltration less likely, and normal bilirubin without obstruction argued against large-duct or hilar disease. AMA-M2 positivity with alkaline phosphatase elevation fulfilled the biochemical and serological criteria for PBC (16), but because pre-treatment antibodies and histology were absent, the temporal relationship to ICI exposure remained unestablished. The fall in cholestatic enzymes after atezolizumab discontinuation and UDCA continuation is consistent with, but does not establish, a contribution from ICI-related biliary injury.

### Central hypothesis: ICI-related biliary injury on a possible underlying primary biliary cholangitis background

AMA-M2 positivity alone does not establish PBC (24), and the significance of PBC-specific antibody positivity with normal alkaline phosphatase remains uncertain (25). Retrospective data suggest that autoimmune or cholestatic liver disease may be tolerated under checkpoint inhibition with monitoring: a review of 22 patients with autoimmune liver disease, including 12 with PBC, showed overall tolerability (17), and case reports describe anti-PD-1 therapy in pre-existing PBC without significant hepatotoxicity (18). These observations are retrospective or case-based and do not establish safety, but they indicate that known autoimmune or cholestatic liver disease is not automatically an absolute contraindication to ICI.

Two explanations remain possible. One is ICI-related biliary injury superimposed on a previously unrecognized PBC background, in which mild pre-treatment cholestasis could represent an underlying autoimmune biliary susceptibility that was aggravated by ICI-related immune activation. The other is *de novo* ICI-related PBC-like injury, reported after ICI exposure with granulomatous hepatitis (19). Both present biochemically as cholestatic injury, and available data do not distinguish them.

As Ruini and colleagues noted, without more complete baseline information “it is not possible to clearly state whether immunotherapy was a mere trigger of a latent autoimmune disease or its primary cause” (19). Serum autoantibody status alone does not reliably predict ICI-related hepatotoxicity (26).

In this patient, pre-treatment cholestatic enzyme abnormalities favor a previously unrecognized PBC background, whereas the absence of pre-treatment AMA-M2 and of histology leaves *de novo* ICI-related PBC-like injury equally possible. Markedly elevated thyroid autoantibodies were consistent with broader immune activation, but their temporal relationship to ICI is uncertain. This favors documenting baseline autoimmunity before ICI exposure and a biliary-specific diagnostic route during therapy.

### Role of imaging and pathology in the evaluation of ICI-associated cholangiopathy

Imaging has a defined but limited role: some patients have negative computed tomography or ultrasound yet biopsy confirms sclerosing cholangitis (27). Two patients with normal magnetic resonance cholangiopancreatography had primary sclerosing cholangitis-like changes on biopsy (28), while another showed common bile duct dilation and wall thickening (29).

Histologically, immune-related sclerosing cholangitis shows periductal fibrosis, cholangitis, and small-duct loss (ductopenia) (28, 30); the French series recommended biopsy for cholestatic-predominant injury after microscopic bile-duct involvement in nearly 40% of patients (11). Negative imaging does not exclude cholangiopathy (27), and abnormal imaging does not confirm it (28).

### Treatment strategy and oncologic outcome: management after ICI-associated cholangiopathy

No uniform guideline exists for ICI-related cholangiopathy; management relies on general immune-related adverse event principles and begins with discontinuation of the suspected ICI. UDCA has shown potential benefit in cholestatic ICI-related liver injury: in 27 patients, UDCA monotherapy produced improvement in 81.5% (median 39.3 days), with 23% relapse after rechallenge (31). For severe or uncertain cases, systematic-review algorithms recommend glucocorticoids first-line and mycophenolate mofetil second-line for refractory disease (32, 33), although the cholestatic phenotype responds poorly to steroids.

In this patient, atezolizumab was discontinued and UDCA continued without immediate high-dose glucocorticoids, and the cholestatic enzymes subsequently fell. Disease control was maintained with dose-intensified consolidative thoracic radiotherapy, furmonertinib directed at the EGFR L858R alteration, and concurrent oral etoposide. These observations describe the course of one patient only and do not establish the feasibility or superiority of this sequence.

Given the solitary contralateral pulmonary metastasis and partial response without new distant disease, thoracic consolidative radiotherapy was selected to intensify local control. Such radiotherapy may benefit selected responding patients with extensive-stage small cell lung cancer (34); however, the 60 Gy in 30 fractions used here was a dose-intensified regimen and should not be interpreted as a uniform standard or as reclassification to limited-stage disease. After a negative brain MRI, PCI was selected following individualized benefit-risk assessment; MRI surveillance is also an evidence-based option (35). No survival benefit from either intervention can be inferred from this single case.

## Limitations

Several limitations should be acknowledged. The evidence rests on a single case and cannot be generalized. Pre-treatment AMA-M2 testing was not performed, so a latent PBC could be neither established nor excluded, and the absence of magnetic resonance cholangiopancreatography, endoscopic retrograde cholangiopancreatography, and liver biopsy left the histologic subtype undefined and the cholestatic phenotype unconfirmed as cholangiopathy. Component-level sequencing was not performed, so the cellular origin of the EGFR mutation could not be assigned to either histologic component, and ICI rechallenge was not attempted, leaving the durability of the biochemical improvement unknown. Above all, the available evidence cannot distinguish a previously unrecognized PBC background from *de novo* ICI-related PBC-like cholangiopathy, nor can it establish how much of the cholestatic injury was attributable to ICI exposure; AMA-M2 positivity favors the former explanation, but the two cannot be separated without pre-treatment serology and histology.

## Conclusion

A 68-year-old man with EGFR L858R-mutant combined small cell lung cancer developed cholestatic liver injury during first-line atezolizumab plus etoposide–platinum, corresponding to CTCAE version 5.0 grade 2. AMA-M2 positivity with pre-treatment cholestatic enzyme abnormalities raised the possibility of an unrecognized primary biliary cholangitis, but without pre-treatment serology, magnetic resonance cholangiopancreatography, or liver biopsy, neither a definite PBC background nor a definite ICI-related cholangiopathy could be established. Liver enzymes improved after atezolizumab discontinuation without high-dose corticosteroids, and oncologic control was maintained. Cholestatic liver injury during immunotherapy warrants evaluation for both autoimmune biliary disease and drug-related biliary injury.

## Patient perspective

Although the treatment process was sometimes difficult, the medical team was always patient and encouraging, which made me feel reassured. The long-term oral treatment and radiotherapy went more smoothly than I had expected, and the side effects remained manageable. My disease is currently relatively stable. I will continue to attend follow-up appointments on time and actively cooperate with further follow-up, and I remain confident about the future.

## Data Availability

The original contributions presented in the study are included in the article/supplementary material. Further inquiries can be directed to the corresponding author.
